# Association between serum uric acid and homocysteine levels among adults in the United States: a cross-sectional study

**DOI:** 10.1186/s12872-023-03586-0

**Published:** 2023-12-08

**Authors:** Jiangsha Wang, Jie Zhou, Zhengping Shao, Xi Chen, Zhenhai Yu, Wenyan Zhao

**Affiliations:** 1 Department of Cardiology, Jiande Hospital of Integrated Traditional Chinese and Western Medicine, Hangzhou, Zhejiang China; 2 Center for General Practice Medicine, General Practice and Health Management Center, Zhejiang Provincial People’s Hospital (Affiliated People’s Hospital, Hangzhou Medical College), Hangzhou, Zhejiang 310014 China; 3 Department of Neurosurgery, De Qing People’s Hospital, Deqing, Zhejiang China

**Keywords:** Uric acid, Homocysteine, Adults, NHANES

## Abstract

**Background:**

Many studies have shown that both elevated serum uric acid (SUA) levels and hyperhomocysteinemia are risk factors for atherosclerosis. However, the relationship between the two has not been thoroughly investigated.

**Objective:**

This study aimed to explore the possible link between SUA levels and homocysteine (Hcy) levels.

**Methods:**

In this cross-sectional study, 17,692 adults aged > 19 years in National Health and Nutrition Examination Survey from 1999 to 2006 were analyzed. Multivariable linear regression analysis was performed to assess the association between SUA and Hcy levels. In addition, smooth curve fitting (penalized spline method) and threshold effect analysis were performed.

**Results:**

Multivariable linear analysis showed that Hcy levels increased by 0.48 µmol/L (β = 0.48, 95%CI: 0.43–0.53) for every 1 mg/dL increase in SUA levels. We found a nonlinear relationship between SUA and Hcy levels. The results of threshold effect analysis showed that the inflection point for SUA levels was 7.1 mg/dL (β = 0.29, 95% CI: 0.23–0.36 and β = 1.05, 95% CI: 0.67–1.43 on the left and right sides of the inflection point, respectively). The *p*-values was less than 0.001 when using the log likelihood ratio test. This nonlinear relationship was also found in both sexes. The inflection point for SUA levels was 5.4 mg/dL in males and 7.3 mg/dL in females, respectively.

**Conclusions:**

This cross-sectional study showed that the SUA levels were positively correlated with Hcy levels. And we found a nonlinear relationship between SUA and Hcy levels.

## Introduction

Serum uric acid (SUA) is the final oxidized product of purine metabolism and is widely used as a biological marker for gout. Epidemiological data have shown that elevated SUA levels are associated with the occurrence and development of various atherosclerosis-related diseases, such as hypertension [1], coronary heart disease (CHD) [2], and stroke [3]. The progression of atherosclerosis caused by SUA may involve the following mechanisms: (i). a reduced production of nitric oxide (NO) which induces endothelial dysfunction [4], decreases the bioavailability of NO, and reduces the dependent vasodilation of the effect [5]; and (ii). stimulation of the vascular endothelial cells by SUA to produce angiotensin II [6].

Homocysteine (Hcy) is a non-essential amino acid derived from methionine metabolism [7]. Hcy has been identified as a common and potent factor in coronary, cerebral, and peripheral atherosclerotic vascular diseases and arteriovenous embolism [8, 9]. Hyperhomocysteinemia (HHcy) is an independent risk factor for peripheral arterial disease [10, 11]. In a cross-sectional study by Tribouilly et al. [12], plasma Hcy levels were positively correlated with thoracic aortic atherosclerosis severity scores and aortic intimal lesion severity. The mechanism by which Hcy causes atherosclerosis includes impairment of endothelial function and reverse cholesterol transport [13].

Hyperuricemia and HHcy have been well studied in relation to cardiovascular disease, and both have an impact on the development of atherosclerosis. However, only a limited number of studies have discussed the relationship between SUA levels and Hcy levels. A recent cross-sectional study showed a positive correlation between Hcy levels and SUA levels among American adolescents aged 12–19 years [14]. Wang et al. [15] found that elevated SUA levels were associated with HHcy in Chinese hypertensive population. While, there are fewer studies on the correlation between SUA levels and Hcy levels in the whole population. Therefore, we used the National Health and Nutrition Examination Survey (NHANES) 1999–2006 database to investigate the possible relationship between SUA levels and Hcy levels among adults in the United States.

## Methods

### Study design and population

Data from the NHANES 1999–2006, a complex, stratified, multi-stage probability sample of the non-institutionalized population in the US, were analyzed. These cross-sectional surveys were conducted at the National Center for Health Statistics (NCHS). More information on the NHANES survey design is available at www.cdc.gov/nchs/nhanes/. The study protocol was approved by the NCHS Ethics Review Committee, and all participants of the NHANES consented to the use of their anonymous information for research purposes.

### Study variables

In this study, SUA levels was the exposure variable. Between 1999 and 2001, Hitachi 704 Multichannel Analyzer (Boehringer Mannheim,) or Roche Hitachi Model 917 were used to measure SUA levels, while Beckman Synchron Lx20 has been in use since 2002. Hcy levels were measured using an Abbott IMx analyzer between 1999 and 2000, an Abbott Homocysteine IMx analyzer in 2001, and an Abbott AxSYM analyzer in 2002. If the covariates altered the regression coefficient by at least 10% when added to the model, they were adjusted as potential confounders. The following potential confounders were included: age, sex, race/ethnicity, education status, alcohol consumption, smoking, physical activity, CHD, hypertension, diabetes, BMI, total polyunsaturated fatty acids, total saturated fatty acids, total protein, dietary fiber, serum vitamin B12, RBC folate, serum folate, and estimated glomerular filtration rate (eGFR). These covariates were adjusted for in the multivariable adjusted model. More information on the covariates analyzed and how the SUA and Hcy levels were measured is available at http://www.cdc.gov/nchs/nhanes/.

### Statistical analysis

The statistical analyses were conducted according to the Centers for Disease Control and Prevention guidelines (https://wwwn.cdc.gov/nchs/nhanes/tutorials/default. Aspx). Significant differences were calculated using the proposed weighting method. Continuous variables are expressed as means (standard error (SE)), and categorical variables as percentages (SE). Weighted chi-square tests (categorical variables) and weighted linear regression analyses (continuous variables) were used to calculate the SUA quartile differences. A multivariable linear regression model was employed to investigate the relationship between SUA and Hcy levels. Three models were constructed as follows: model 1, no covariates were adjusted; model 2, minimally adjusted model for age and sex; and model 3, adjusted for the potential confounders. To evaluate the nonlinear properties of SUA and Hcy levels, smooth curve fitting (penalized spline method) were used. When a non-linear correlation was noted between SUA and Hcy levels, a two-piecewise linear regression model was used to calculate the inflection point of SUA on Hcy. The likelihood-ratio test was used for nonlinearity. A subgroup analysis was performed using a stratified linear regression analysis.

All tests were two-sided and statistical significance was set at *p* < 0.05. All analyses were performed using the statistical software packages R (http://www.R-project.org, The R Foundation), EmpowerStats (http://www.empowerstats.com, X&Y Solution, Inc., Boston, MA) and Free Statistics software.

## Results

### Participant selection

**Fig. 1:**
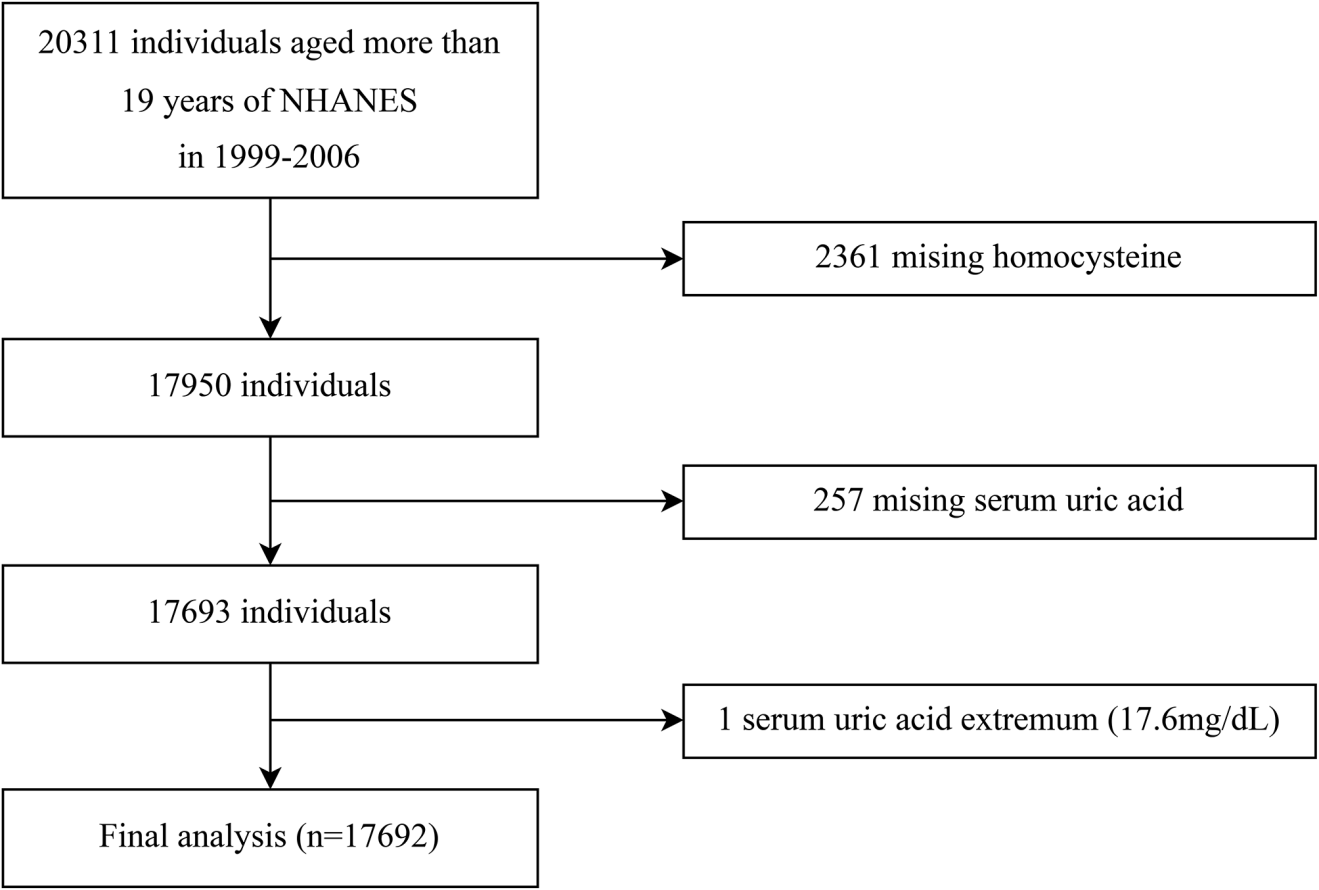
**Flow chart of participants**

A total of 20,311 participants aged > 19 years were selected from the NHANES 1999–2006 database. After excluding those with missing Hcy (n = 2361) and SUA values (n = 257) and with SUA levels > 17.6 mg/dL (n = 1), 17,692 eligible participants were included in the final analysis (Fig. 1).

### Participant characteristics

The participants’ mean age was 46.07 years, and approximately 48% were male. According to the SUA quartiles, the selected participants’ social demographic characteristics and the weighted distribution of the other characteristics were shown in Table 1. The SUA quartile ranges for Q1 to Q4 were < 4.2, 4.2–5.1, 5.1–6.2, and ≥ 6.2 mg/dL, respectively. Compared with participants in the other groups, those in Q4 with higher SUA levels, had higher BMI, SBP, DBP, TMFA, TPFA, TSFA, total fat intakes, protein intakes, energy intake and Hcy levels. However, they had lower vitamin B6 and folic acid supplement, serum folate, serum vitamin B12 and eGFR. Participants in the Q4 group were slightly older, more male, tended to smoke, consume alcohol, live a sedentary lifestyle, and have CHD, hypertension and diabetes.

**Table 1:** Characteristics of study participants

Characteristics	Serum uric acid, mg/dL					
	Total	Q1 (< 4.2)	Q2 (4.2–5.1)	Q3 (5.1–6.2)	Q4 (≥ 6.2)	*P* value
N^a^	17,692	4333	4040	4766	4553	
age,years	46.07 (0.34)	43.07 (0.51)	45.93 (0.45)	46.92 (0.44)	48.12 (0.65)	< 0.001
Male,%	48.02 (0.39)	12.04 (0.74)	37.05 (1.33)	62.05 (0.89)	77.03 (0.96)	< 0.001
**Race/ethnicity, %**						
Non-Hispanic White	71.64 (1.85)	69.62 (2.20)	71.73 (1.83)	71.46 (2.02)	73.6 (2.03)	0.025
Non-Hispanic Black	10.14 (1.17)	10.84 (1.43)	8.98 (1.01)	10.06 (1.34)	10.58 (1.23)	0.886
Mexican American	7.07 (0.87)	7.86 (0.96)	7.11 (1.00)	7.1 (0.97)	6.25 (0.79)	0.013
Other Hispanic	6.89 (1.67)	7.62 (1.77)	7 (1.62)	7.47 (2.16)	5.53 (1.59)	0.105
Other Race/Ethnicity	4.27 (0.60)	4.06 (0.67)	5.18 (0.90)	3.91 (0.60)	4.03 (0.89)	0.611
**Education,%**						
Less than high school	21.58 (0.83)	19.77 (1.57)	22.39 (1.45)	21.59 (1.16)	22.54 (0.99)	0.219
High school	25.55 (0.97)	22.72 (1.20)	25.14 (1.79)	26.41 (1.39)	27.67 (1.20)	< 0.001
More than high school	52.86 (1.40)	57.51 (2.00)	52.47 (2.28)	52 (1.66)	49.78 (1.38)	0.002
**Alcohol consumption, g/day**						
<5	83.56 (0.62)	91.67 (0.77)	85.79 (0.99)	80.37 (1.22)	77.83 (1.39)	< 0.001
≥5	16.44 (0.62)	8.33 (0.77)	14.21 (0.99)	19.63 (1.22)	22.17 (1.39)	< 0.001
**Smoking,%**						
Never	50.43 (1.29)	56.4 (1.72)	51.05 (1.99)	48.26 (1.97)	46.52 (1.48)	< 0.001
Current	25.32 (0.96)	20.08 (1.64)	23.69 (1.16)	26.54 (1.35)	30.4 (1.31)	< 0.001
Former	24.25 (0.87)	23.53 (1.23)	25.26 (1.59)	25.2 (1.27)	23.09 (1.26)	0.801
**Physical activity,%**						
Sedentary	21.55 (1.10)	21.67 (1.21)	20.72 (1.68)	19.59 (1.29)	24.18 (1.40)	0.125
Low	27.41 (0.86)	26.64 (1.22)	28.47 (1.71)	26.87 (1.50)	27.73 (1.35)	0.804
Moderate	18.91 (0.72)	21.07 (1.37)	18.61 (1.14)	19.04 (1.05)	16.96 (0.95)	0.011
High	32.14 (0.94)	30.61 (1.50)	32.21 (1.87)	34.5 (1.32)	31.12 (1.53)	0.602
**Comorbidities,%**						
CHD	6.88 (0.41)	3.82 (0.51)	5.41 (0.43)	6.35 (0.62)	11.53 (0.99)	< 0.001
Hypertension	40.42 (0.87)	26.88 (1.40)	36.87 (1.50)	42.79 (1.42)	53.71 (1.23)	< 0.001
Diabetes	7.92 (0.38)	6.4 (0.63)	8.03 (0.59)	6.61 (0.59)	10.53 (0.77)	< 0.001
**Physical examination**						
BMI, kg/m^2^	28 (0.14)	25.74 (0.17)	27.43 (0.20)	28.41 (0.18)	30.20 (0.25)	< 0.001
Mean systolic, mmHg	118.03 (0.37)	113.51 (0.63)	117.22 (0.62)	119.27 (0.48)	122.12 (0.47)	< 0.001
Mean diastolic, mmHg	71.69 (0.28)	68.94 (0.44)	71.53 (0.48)	72.35 (0.36)	73.94 (0.44)	< 0.001
**Dietary**						
TMFA intake, gm	30.82 (0.26)	27.61 (0.40)	29.78 (0.52)	32.25 (0.54)	33.24 (0.48)	< 0.001
TPFA intake, gm	16.96 (0.17)	15.62 (0.28)	16.47 (0.27)	17.58 (0.42)	18.01 (0.27)	< 0.001
TSFA intake, gm	27.00 (0.25)	24.4 (0.38)	26.34 (0.58)	28.09 (0.47)	28.87 (0.42)	< 0.001
Total fat intake, gm	82.47 (0.63)	74.51 (0.97)	80.19 (1.37)	85.94 (1.36)	88.32 (1.07)	< 0.001
Protein intake, gm	81.92 (0.66)	72.05 (0.86)	79.84 (1.45)	85.08 (1.14)	89.63 (0.99)	< 0.001
Dietary fiber intake, gm	15.93 (0.25)	15.23 (0.27)	15.73 (0.36)	16.59 (0.45)	16.09 (0.31)	0.008
Energy intake, kcal	2207.89 (15.29)	1989.29 (23.48)	2129.78 (31.88)	2290.98 (32.35)	2393.79 (23.74)	< 0.001
**Supplement use**						
Vitamin B6, mg	5.12 (0.41)	6.28 (0.65)	4.87 (0.70)	5.3 (0.91)	4.09 (0.52)	0.018
Vitamin B12, mcg	20.83 (3.96)	16.7 (2.73)	21.69 (3.27)	14.34 (1.92)	30.35 (13.46)	0.411
Folic acid, mcg	136.87 (8.66)	191.09 (31.37)	130.26 (8.07)	123.53 (8.31)	105.99 (5.11)	0.008
**Laboratory data**						
Homocysteine, µmol/L	8.54 (0.06)	7.25 (0.10)	8.03 (0.12)	8.69 (0.10)	10.04 (0.13)	< 0.001
RBC folate, ng/mL	309.54 (3.92)	308.95 (5.52)	308.24 (4.84)	303.5 (4.90)	317.21 (4.93)	0.224
Serum folate, ng/mL	15.03 (0.22)	15.25 (0.38)	15.35 (0.32)	14.9 (0.24)	14.68 (0.30)	0.047
Serum Vitamin B12, pg/mL	542.17 (19.49)	540.63 (21.48)	610.18 (73.34)	540.35 (25.48)	485.37 (5.94)	0.029
eGFR, ml/min per 1.73 m^2b^	100.53 (0.49)	108.95 (0.72)	102.27 (0.73)	98.4 (0.68)	93.33 (0.71)	< 0.001

Note: Continuous variables were presented as mean (SE), calculated by weighted linear regression model. Categorical variables were presented as number (%) (SE), calculated by weighted chi-square test

^a^Unweighted number of observations in dataset

^b^Estimated using the CKD-EPI equation. eGFR = 141 × min(Scr/κ, 1)^α^ × max(Scr/κ, 1)^−1.209^ × 0.993^Age^ × 1.018 [if female] × 1.159 [if black], where Scr is serum creatinine, κ is 0.7 for females and 0.9 for males, α is -0.329 for females and − 0.411 for males, min indicates the minimum of Scr/κ or 1, and max indicates the maximum of Scr/κ or 1

Abbreviations: CHD, coronary heart disease; BMI, body mass index; RBC, red blood cell; TMFA, total monounsaturated fatty acids; TPFA, total polyunsaturated fatty acids; TSFA, total saturated fatty acids; eGFR, estimated glomerular filtration rate

### Association between SUA levels and hcy levels

The association between SUA levels and Hcy levels were presented in Table 2. In the non-adjusted model, every 1 mg/dL increase in SUA levels was associated with a 1.03 µmol/L increase in Hcy levels (β = 1.03, 95% confidence interval (CI): 0.99–1.08, *p* < 0.001). In the multivariable-adjusted model, this trend was consistent. Every 1 mg/dL increase in SUA levels was associated with an increase in Hcy levels of 0.48 µmol/L (β = 0.48, 95% CI: 0.43–0.53, *p* < 0.001). To test the robustness of the results, SUA was treated as a categorical variable (quartiles) in an additional analysis, which showed no changes in the trend (Table 2).

**Table 2:** Association of homocysteine with serum uric acid

SUA, mg/dL	Hcy, µmol/L, β (95% CI), *P*-value		
	Non-adjusted model	Minimally adjusted model	Multivariable adjusted model
Per 1-mg/dL increment	1.03 (0.99, 1.08) < 0.001	0.72 (0.67, 0.76) < 0.001	0.48 (0.43, 0.53) < 0.001
Quartile			
Q1 (< 4.2)	Ref	Ref	Ref
Q2 (4.2–5.1)	1.13 (0.93, 1.32) < 0.001	0.49 (0.30, 0.67) < 0.001	0.12 (-0.06, 0.31) 0.186
Q3 (5.1–6.2)	2.07 (1.88, 2.25) < 0.001	1.03 (0.84, 1.22) < 0.001	0.49 (0.30, 0.69) < 0.001
Q4 (≥ 6.2)	3.63 (3.44, 3.81) < 0.001	2.26 (2.07, 2.46) < 0.001	1.34 (1.13, 1.56) < 0.001
*P* for trend	< 0.001	< 0.001	< 0.001

Non-adjusted model:no covariates were adjusted

Minimally adjusted model:adjusted for age, sex

Multivariable adjusted model: adjusted for age, sex, race/ethnicity, education status, alcohol consumption, smoking, physical activity, coronary heart disease, hypertension, diabetes, BMI, total polyunsaturated fatty acids, total saturated fatty acids, total protein, dietary fiber, serum vitamin B12, RBC folate, serum folate, estimated glomerular filtration rate

Abbreviations: SUA, serum uric acid, Hcy, homocysteine; 95% CI, 95% confidence interval

### Nonlinear relationship between SUA levels and hcy levels

**Fig. 2:**
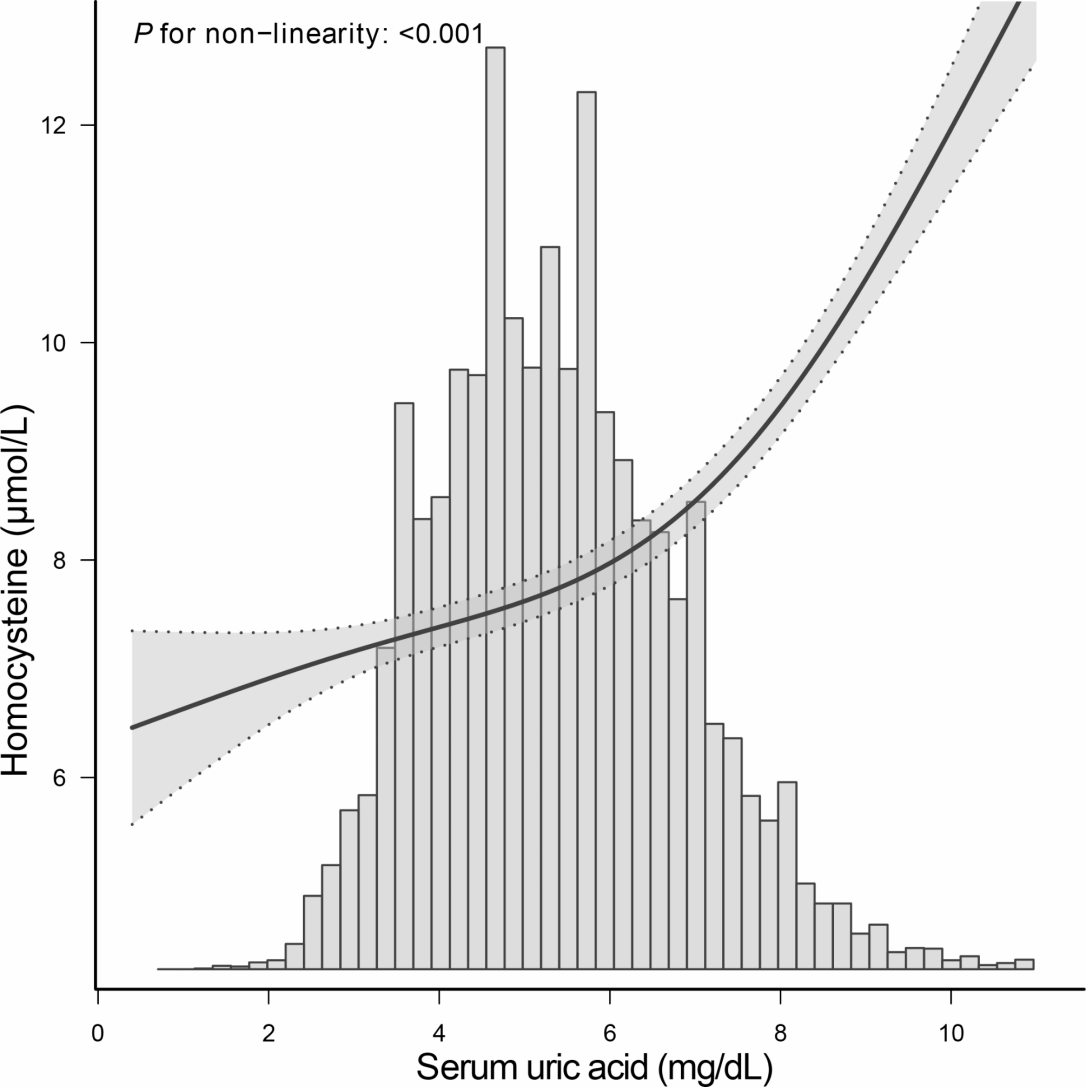
**Dose-response relationship between serum uric acid levels and homocysteine levels.** Analyses were adjusted for age, sex, race/ethnicity, education status, alcohol consumption, smoking, physical activity, coronary heart disease, hypertension, diabetes BMI, total polyunsaturated fatty acids, total saturated fatty acids, total protein, dietary fiber, serum vitamin B12, RBC folate, serum folate, estimated glomerular filtration rate

**Fig. 3:**
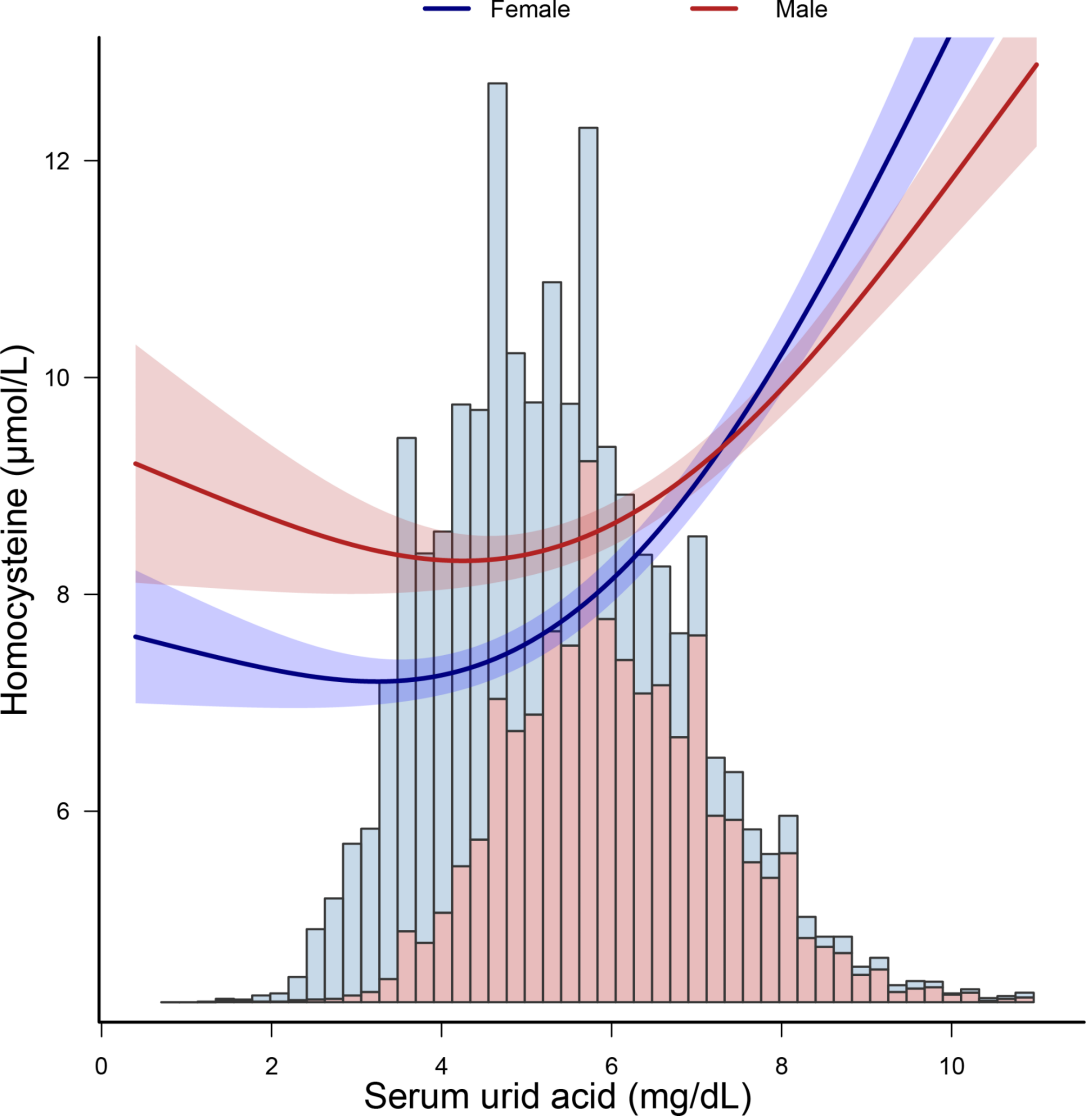
**Dose-response relationship between serum uric acid levels and homocysteine levels stratified by sex.** Analyses were adjusted for age, race/ethnicity, education status, alcohol consumption, smoking, physical activity, coronary heart disease, hypertension, diabetes BMI, total polyunsaturated fatty acids, total saturated fatty acids, total protein, dietary fiber, serum vitamin B12, RBC folate, serum folate, estimated glomerular filtration rate

Smooth curve fitting were used to show the dose-response relationship between SUA and Hcy levels. The multivariable adjusted smooth curve fitting showed a nonlinear association between SUA and Hcy levels (*p* < 0.001, Fig. 2). The nonlinear *p*-value was less than 0.001, which indicated a nonlinear relationship between SUA and Hcy levels. Since sex is a known confounder of the association between SUA and Hcy, we examined the nonlinear relationship between SUA and Hcy levels stratified by sex. The sex subgroup analysis generated similar findings (Fig. 3).

### Threshold effect analysis of SUA levels on hcy levels

Owing to the nonlinear correlation between SUA and Hcy levels, a two-piecewise linear regression model was used to calculate the inflection point of SUA on Hcy levels (Table 3). In the total population, the inflection point for SUA levels was 7.1 mg/dL. For every 1 mg/dl increase in SUA levels on the right side of the inflection point compared to the left side of the inflection point, the increase in Hcy levels was higher [β = 0.29, 95% CI (0.23–0.36) vs. β = 1.05, 95% CI (0.67–1.43)]. The *p*-values was less than 0.001 when using the log likelihood ratio test. A nonlinear relationship between SUA and Hcy levels was also supported in both males and females, but the inflection point value of SUA was different in males and females. The inflection point for SUA was 5.4 mg/dL in males and 7.3 mg/dL in females, respectively (Table 3).

**Table 3:** Threshold effect analysis of serum uric acid on homocysteine by sex

	Hcy, µmol/L	
	β (95% CI)^b^	*P*-value
Total		
SUA, mg/dL	0.48 (0.43, 0.53)	< 0.001
Inflection point^1^		
< 7.1	0.29 (0.23, 0.36)	< 0.001
> 7.1	1.05 (0.67, 1.43)	< 0.001
*P* for log likelihood ratio test		< 0.001
Male		
SUA, mg/dL	0.45 (0.37, 0.52)	< 0.001
Inflection point^a^		
< 5.4	-0.16 (-0.40, 0.08)	0.193
> 5.4	0.65 (0.53, 0.77)	< 0.001
*P* for log likelihood ratio test		< 0.001
Female		
SUA, mg/dL	0.53 (0.45, 0.61)	< 0.001
Inflection point^1^		
< 7.3	0.35 (0.26, 0.43)	< 0.001
> 7.3	1.68 (0.51, 2.86)	0.005
*P* for log likelihood ratio test		< 0.001

^a^Fitting model by two-piecewise linear regression model

^b^Adjusted: age, race/ethnicity, education status, alcohol consumption, smoking, physical activity, coronary heart disease, hypertension, diabetes, BMI, total polyunsaturated fatty acids, total saturated fatty acids, total protein, dietary fiber, serum vitamin B12, RBC folate, serum folate, estimated glomerular filtration rate

Abbreviations: SUA, serum uric acid, Hcy, homocysteine; 95% CI, 95% confidence interval

### Subgroup analysis

**Fig. 4:**
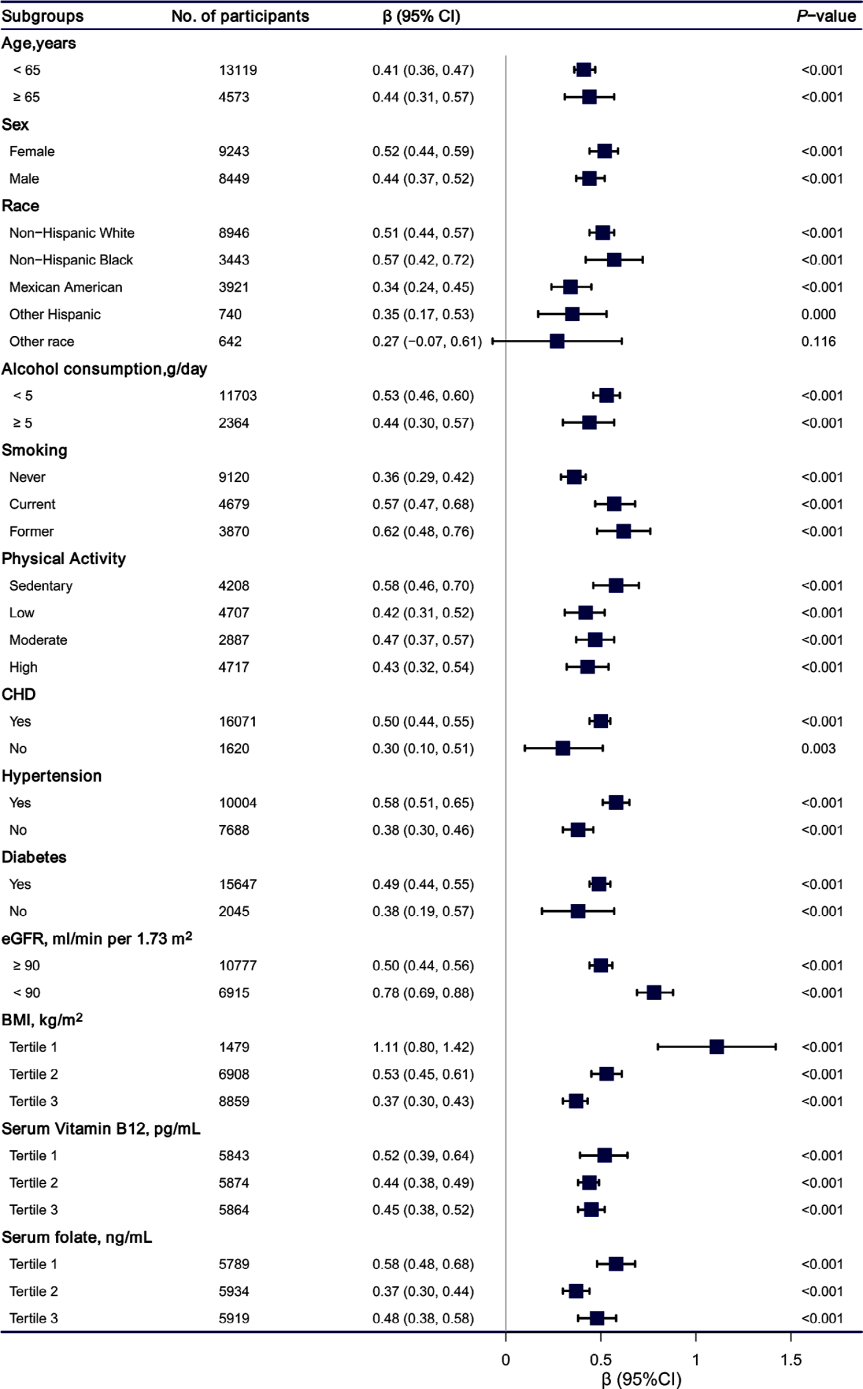
**Association between serum uric acid levels and homocysteine levels according to subgroup.** Analyses were adjusted for age, sex, race/ethnicity, education status, alcohol consumption, smoking, physical activity, coronary heart disease, hypertension, diabetes BMI, total polyunsaturated fatty acids, total saturated fatty acids, total protein, dietary fiber, serum vitamin B12, RBC folate, serum folate, estimated glomerular filtration rate. Abbreviations: CHD, coronary heart disease; eGFR, estimated glomerular filtration rate; 95% CI, 95% confidence interval

The results of the subgroup analysis of the association between SUA and Hcy levels were presented in Fig. 4. The association between SUA and Hcy levels in the stratified analysis was consistent with the findings of the multivariable linear regression analysis.

## Discussion

SUA levels was positively correlated with Hcy levels after adjusting for other covariates. Each 1 mg/dL increase in SUA was associated with a 0.48 µmol/L increase in Hcy (β = 0.48, 95% CI: 0.43–0.53). We found a nonlinear relationship between SUA and Hcy levels. Additionally, the subgroup analysis showed that the correlation between SUA and Hcy levels was stronger in those with lower vitamin B12 and folic acid, less alcohol consumption and lower eGFR.

Previous studies, conducted among patients with specific diseases such as diabetes [16] and cognitive impairment [17], have reported a positive correlation between SUA and Hcy levels. Hyperuricemia was also positively associated with HHcy in a study by Cohen [18], which is consistent with the findings of this study. Additionally, here, the relationship between SUA and Hcy levels was nonlinear after adjusting for many covariables using a two-piecewise linear regression model stratified by sex. Further, the inflection point was 5.4 mg/dL in males and 7.3 mg/dL in females.

Adenosine molecules may be responsible for the relationship between hyperuricemia and HHcy. In the methionine-Hcy cycle [19], methionine is converted to S-adenosyl-l-homocysteine (SAH), which is reversibly catabolized to adenosine and Hcy by SAH hydrolase [20]. Under normal conditions, SAH hydrolase also recirculates adenosine and Hcy back into SAH [21]. However, this requires a 1:1 stoichiometric ratio of Hcy to adenosine. Therefore, if one of these substances is missing, the circulation kinetics will be affected, leading to the accumulation of the other. In a hypoxic or ischemic state, there is an increased release of adenosine from the circulation. During purine metabolism, this is quickly degraded to SUA, resulting in elevated SUA levels and Hcy accumulation.

Folic acid and vitamin B12 are important coenzymes of Hcy metabolism. If deficiencies of folic acid and vitamin B12 exist, the conversion of Hcy is blocked and, subsequently, Hcy accumulates in the body. Several studies have shown that Hcy levels are negatively correlated with folic acid [22] and vitamin B12 levels [23, 24]. Here, in the subgroup analysis, the SUA levels increased more per 1 mg/dL of elevated Hcy in the low-folate and vitamin B12 groups than in the high-folate and vitamin B12 groups. It is important to consider that high SUA levels may exacerbate the impaired Hcy metabolism among people with low folic acid and vitamin B12 levels.

In the subgroup analysis, participants who drank < 5 g of alcohol per day had smaller increases in Hcy levels when SUA increased by 1 mg/dL, compared with those who drank > 5 g of alcohol per day. This may be owing to alcohol metabolism resulting in elevated adenosine levels [25]. Since SAH hydrolase recirculates adenosine and Hcy back to SAH in the methionine-Hcy cycle, Hcy clearance increases significantly in the presence of elevated adenosine levels.

Many studies investigating SUA and kidney disease [26] have shown that elevated SUA levels may lead to renal insufficiency, resulting in a decreased eGFR. Renal function plays an important role in Hcy metabolism, as decreased renal function equates to poorer clearance of Hcy [26, 27]. Similarly, the subgroup analysis showed a more pronounced increase in Hcy levels for every 1 mg/dL increase in SUA levels among those with low eGFR compared with those with high eGFR.

There is growing evidence suggesting that hyperuricemia and HHcy play a pivotal role in the development of atherosclerosis. We observed a direct correlation between SUA and Hcy levels, and this held true for both males and females. Numerous epidemiologic reports confirm that HHcy is an independent risk factor for cardiovascular diseases. Although combination therapy with folic acid and B vitamins has been shown to substantially reduce Hcy levels, the results of randomized placebo-controlled clinical trials examining the impact of combination therapy with folic acid and B vitamins on the outcomes of these diseases have yielded mixed but generally were not satisfactory [28]. Thus, although there is no doubt that folic acid and B vitamin supplementation reduce Hcy levels, further research is needed to determine which types of patients would benefit most from such treatments. From our study, it is clear that elevated SUA levels is associated with HHcy, suggesting that the benefit of combined treatment with folic acid and B vitamin supplements in reducing the risk of cardiovascular disease in patients with hyperuricemic could be further investigated.

This study has several limitations that should be considered when interpreting the results. First, owing to the nature of cross-sectional studies, determining the causal relationship between SUA and Hcy is difficult. Future large prospective studies or bidirectional Mendelian randomization studies need to be performed to determine a causal relationship between the two. Second, since the study participants were adults from the United States, the generalizability of the findings to other populations may be limited. Third, as an observational study, uncontrolled confounding or reverse causality could not be completely excluded.

## Conclusion

In conclusion, SUA levels was positively correlated with Hcy levels among adults in the United States and the associations were more significant among those with low folic acid levels, vitamin B12 levels, alcohol consumption, and eGFR. Further investigation using a prospective cohort study comparing SUA and Hcy levels is necessary to confirm these findings.

## Data Availability

The NHANES datasets are publicly available at:www.cdc.gov/nchs/nhanes/.

## Abbreviations

SUA: Serum uric acid
Hcy: Homocysteine
HHcy: Hyperhomocysteinemia
NO: Nitric oxide
NCHS: National Center for Health Statistics
NHANES: National Health and Nutrition Examination Survey
SAH: S-adenosyl-l-homocysteine
SE: Standard error
eGFR: Estimated glomerular filtration rate
